# Criminal Abortion and Infanticide in Atlanta

**Published:** 1884-10

**Authors:** 


### CRIMINAL ABORTION AND INFANTICIDE IN ATLANTA.

We avail ourselves of data brought to our attention since penning our remarks upon criminal abortion and infanticide, to urge still further the consideration of their enormity by the authorities of our city.

Upon examining the records for the year 1883, it is ascertained that there were eleven dead bodies of male and female new born infants, among whites and blacks, making nearly an average of one case of infanticide for each month of the year. The origin of these deaths could not be traced to the perpetrators, but the circumstances by which they were surrounded led to the conclusion that the children were the fruits of illicit intercourse, whose parents sought this mode to conceal their departure from the path of virtue.

During the current year, during four months, from the 22d of April to the 16th of August, there were six new-born children, whose dead bodies were found in the city, to which no clue could be obtained, but evidently the work of those who seek to conceal one crime by committing another.

The outrageous features of this clandestine murder may appear in its proper light if we suppose that in every month of the year the dead body of some unknown man or woman is found hid away in some dark corner of the streets of Atlanta and no vestige can be discovered of the home of these murdered men and women, nor any trace of the perpetrators of this secret midnight crime.

The only thing known in regard to these murdered strangers is that their dead bodies have been in some clandestine manner placed by unknown hands in some out-of-the-way place to be discovered by some person, or picked up by the police in their rounds through the dark alleys of the city. Every good citizen would doubtless feel great concern for the public safety if such a number of inexplicable deaths were brought to their knowledge, yet six innocent and helpless little children have been coolly and deliberately murdered during four months of this present year in our midst, and their dead bodies cast upon our streets, while scarcely a word of complaint or an expression of surprise greets our ears.

If it is right and proper for mothers to strangle their new-born babes, by parity of reasoning, parents may murder their children of any age, without compunction of conscience; nor can we discover any difference between this class of murderers and those who go out upon the highways with pistols and bludgeons to kill whomsoever they meet.

Those who ignore criminal abortion amongst us may in like manner deny the evidences of a coroner's inquest upon the cast-away infants. But still, facts are stubborn realities which stare us in the face at every turn, and we cannot, if we would, set them aside. They will not be down at our bidding, and it behooves every good citizen to see to it that the existing laws against criminal abortion and infanticide are properly enforced, and we appeal to every respectable physician to support the authorities.

